# Atypical Presentation of Varicella-Zoster Virus Encephalitis: A Case Report

**DOI:** 10.7759/cureus.68926

**Published:** 2024-09-08

**Authors:** Norah T Al-Muwallad, Ahmed Al-Dhahi, Hanan K Aljaidi, Maram Al-balawi

**Affiliations:** 1 Neurology, King Fahad Specialist Hospital, Tabuk, SAU; 2 Neurology, Prince Sultan Military Medical City, Riyadh, SAU

**Keywords:** brain infection, ear pain, encephalitis, fatal disease, varicella-zoster virus, vzv

## Abstract

The varicella-zoster virus (VZV) is a neurotrophic alpha herpesvirus that only affects humans. Once infected (often in childhood), VZV causes varicella (chickenpox) before becoming dormant in the cranial nerve (CN) and dorsal root ganglia. It can reactivate after a period of time, resulting in zoster (shingles), which is occasionally followed by post-herpetic neuralgia. This case highlights a patient who presented with vague ear pain and multiple CN palsy, including CN VIII, IX, and X, preceded by a common cold symptom one week ago. Shortly after, he developed severe pain in his left ear and sought medical care at an ENT clinic. The diagnosis was lymphadenopathy, and he received pain medication and a single dose of antibiotics. The patient was conscious, alert, and oriented. He had no fever with normal WBC. Clinical examination revealed multiple CN palsies. Neuroimaging showed normal study. To address potential bacterial infection, the patient was given vancomycin and ceftriaxone as well as acyclovir after a lumbar puncture was performed. The CSF analysis revealed elevated lymphocytes and VZV DNA was detected in the CSF by using polymerase chain reaction. This is an atypical presentation of VZV encephalitis as the patient presented mainly with ear pain. The neurological complications, including CN palsies related to active CNS varicella-zoster infection, and meningeal involvement were clinically improved with empirical medications. The CSF analysis confirmed the diagnosis. Early diagnosis and treatment with antiviral medication are key to optimizing clinical outcomes.

## Introduction

The varicella-zoster virus (VZV) is a neurotrophic alpha herpes virus that only affects humans. Once infected (often in childhood), the VZV causes varicella (chickenpox) before becoming dormant in the cranial nerve (CN) and dorsal root ganglia. It can reactivate after a period, resulting in zoster (shingles), which is occasionally followed by post-herpetic neuralgia [1]. Reactivation typically happens in elderly people or in those who have impaired immune systems. When newly infected or later if reactivation takes place, VZV can produce a wide range of central nervous system (CNS) signs, including encephalitis, cerebellitis, meningitis, vasculitis, stroke, and polyneuropathy [2]. Shingles are the most typical manifestation of the VZV in adult patients. A painful vesicular eruption that is exclusive to one dermatome of the body is known as shingles. The involvement of the CNS, which can result in encephalitis, is one of the possible side effects of this illness [3]. Immunocompromised patients are more likely to experience this consequence [4]. An altered state of awareness, vomiting, and possibly seizures are symptoms of VZV encephalitis. In this case, the patient had hallucinations, changes in mental status, and vomiting [5]. Often, these symptoms are seen as the adverse effects of valacyclovir administered at a too low renal dosage. The death rate from VZV encephalitis is around 15% in immunocompetent individuals and practically 100% in immunosuppressed patients if the patient is left untreated, especially if the liver and lung are also affected [6]. In encephalitis caused by the VZV, CSF examination often reveals lymphocytic pleocytosis and protein increase, both of which were seen in this instance. VZV is verified by positive polymerase chain reaction (PCR) results in the CSF. However, CSF anti-VZV antibodies cannot be employed as a sole diagnostic tool for VZV-related neurological disorders [7].

## Case presentation

**Figure 1:**
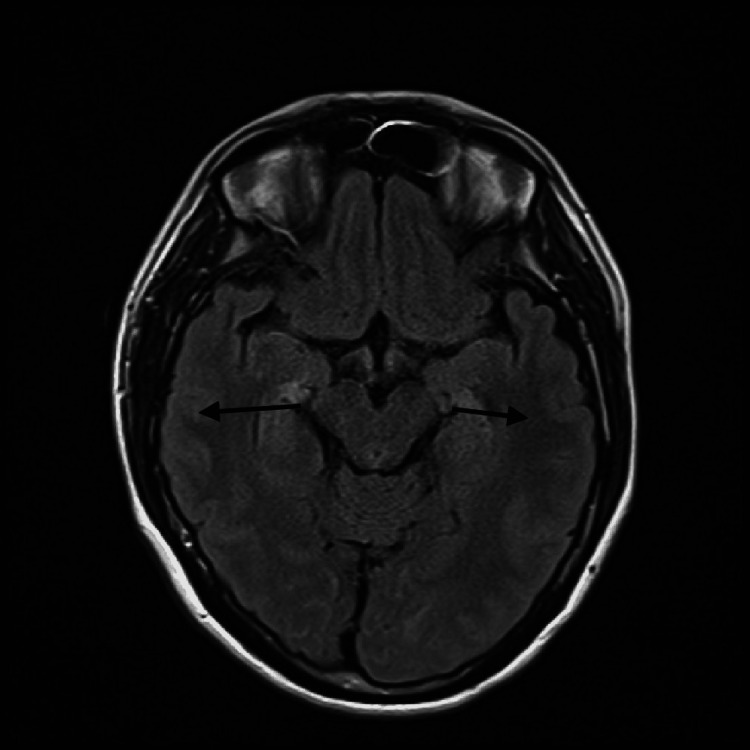
MRI of the brain with contrast, fluid-attenuated inversion recovery (FLAIR) sequence showing normal grey-white interface with no area of abnormal signal intensity, no evidence of mass lesion, no area of abnormal enhancement lesion, and no gross vascular abnormality.

We report a case of a 25-year-old Saudi male, smoker, unemployed patient who was medically free, as he did not take any medication and was not diagnosed with any disease. He had a one-week history of sore throat, runny nose, sneezing, and coughing before he developed severe left ear pain. He went to an outside ENT clinic and was diagnosed with lymphadenopathy and discharged with analgesics and one dose of antibiotics. Three days later, the patient presented to the emergency department complaining of the inability to swallow solids and liquids, a change in the quality of voice, and an unbalanced gait. He had no skin rash or ear rash, no headache, no hearing loss, no abnormal movement, no change in behavior, and a negative history of chickenpox infection. The patient was vaccinated with varicella-zoster vaccine. The patient was conscious, alert, and oriented to time, place, and person. He was afebrile. His pupils were reactive and symmetrical bilaterally, with normal size and shape and visual acuity of 6/6 with preserved color vision and normal extra-ocular movement but he had bilateral vertical and horizontal nystagmus. No tongue deviation was noted during protrusion or fasciculation. No sensory abnormality was noted in the face and body. He had a left-side weakness as he could not resist well, with an ataxic gait and impaired gag reflex. He had no hearing problem, no facial weakness, no neck rigidity, negative meningeal signs, and no lymph node enlargement. The brain magnetic resonance imaging with contrast was negative for an abnormal enhancement, as shown in Figure 1. He was started on empiric antibacterial coverage with vancomycin and ceftriaxone as well as acyclovir after a lumbar puncture was performed. Cerebrospinal fluid (CSF) analysis (Table 1) showed lymphocytic pleocytosis with 60 white blood cells, 97% lymphocytes, 3% neutrophils, glucose at 3.20 mmol/L, and protein at 318 mg/l. A CSF PCR was negative for herpes simplex virus 1 and 2 but VZV DNA was detected. HIV-1/2 antigen/antibody was nonreactive and HIV-1/2 RNA was not detected as well. Antibiotics were stopped and acyclovir 10 mg/kg intravenously three times daily was continued for 21 days. The patient improved clinically and was discharged home with a follow-up at the outpatient neurology clinic.

**Table 1:** CSF analysis of the patient showed high WBC, mainly lymphocytes, with normal glucose, protein, LDH, and lactate, indicating CNS viral infection. LDH: lactate dehydrogenase.

Test name	Patient's result	Normal range (SI unit)
CSF protein	318 mg/l	150-450 mg/L
CSF glucose	3.20 mmol/L	2.77-4.44 mmol/L
CSF lactate	1.89 mmol/L	1.1-2.4 mmol/L
CSF LDH	21.0 U/L	0-40 U/L
CSF WBC	60 cell/ul (97% lymphocytes and 3% neutrophils)	0-5 cell/ul
CSF RBC	0	0 cell/ul

## Discussion

VZV, commonly known as the chickenpox virus, has emerged as a prominent agent associated with various neurological complications, such as encephalitis, meningitis, and myelitis. Studies have indicated that VZV accounted for a substantial 29% of all confirmed or probable etiologic agents in these cases, highlighting its significant contribution to neurological viral infection [8].

In comparison with other viral pathogens, VZV has gained considerable recognition for its role in CNS infections. Herpes simplex virus (HSV) and certain enteroviruses both contributed 11% of the cases, while influenza A virus accounted for 7%. These data imply that VZV has emerged as a dominant player in CNS infections, surpassing other well-known viral agents [8]. Before the implementation of the universal varicella vaccination (UVV), the occurrence of varicella complications among a group of 3,802 patients in Saudi Arabia stood at 1.50%. The frequently observed complications included infections of the skin and soft tissue (0.50%), pneumonia (0.42%), bacteremia (0.16%), encephalitis and cerebellitis (0.11%), as well as myositis and necrotizing fasciitis (0.11%) [9]. The usual symptoms of herpes simplex virus encephalitis (HSVE) commonly include a rise in body temperature, aches in the head, a state of disorientation, and either focal or widespread seizures. Approximately 90% of individuals affected by HSVE typically experience fever and abnormal mental state as the foremost indications. Other common signs and symptoms encompass vomiting, queasiness, pseudomeningitis, and seizures, which affect around 50% to 60% of patients. Additionally, focal neurological impairments are observed in approximately 30% to 50% of cases [10]. To diagnose VZV infection, a combination of clinical history, brain scans (CT or MRI), and CSF examination is required. The CSF examination involves using microscopy, biochemical analysis, and DNA PCR to check for the presence of VZV DNA.

The DNA PCR test for VZV in the CSF is considered the most reliable, with a sensitivity of 94-98% and specificity of 98-100% [11]. This instance of VZV encephalitis highlighted a situation where a patient exhibited atypical clinical symptoms and normal brain imaging results, but their CSF examination showed abnormalities. Nevertheless, initiating early treatment with intravenous acyclovir, based on assumptions, resulted in the patient's complete neurological recovery, which is remarkable considering VZV is typically a highly fatal disease if it affects the CNS and is left without treatment.

## Conclusions

This is one of the rare presentations of a dangerous disease. The patient exhibited neurological complications, including cranial nerve palsies, atypical clinical symptoms, and normal neuroimaging results, but his CSF examination showed abnormalities. In this case, initiation of early treatment with acyclovir, based on assumptions, led to the patient's complete neurological recovery. VZV infection has a mortality rate of 12-15%, but it can be higher in immunocompromised patients if not treated with antiviral agents. Early diagnosis and treatment are key to optimizing good clinical outcomes.
